# Can a difference in socioeconomic levels influence executive functioning in a Tunisian population?

**DOI:** 10.1192/j.eurpsy.2025.1976

**Published:** 2025-08-26

**Authors:** M. Ben Hmida, D. Le Gall, T. Bellaj, R. Ben Rejeb

**Affiliations:** 1 Psychology, Université d’Angers, Angers, France; 2 Psychology, Université de Tunis, Tunis, Tunisia

## Abstract

**Introduction:**

Poverty’s impact on mental health and quality of life is well documented. Studies have shown that the high stress levels associated with poverty increase the risk of mental illness. Moreover, cognitive abilities seem to be influenced by a low socioeconomic level. Conversely, individuals who benefit from a better socioeconomic situation since childhood and throughout adulthood are more likely to succeed academically and professionally.

**Objectives:**

This paper examines the relationship between socioeconomic status and executive functioning in healthy Tunisian adults. It aims to determine if socioeconomic differences influence executive processes.

**Methods:**

We conducted an experimental study with 95 Tunisian adults (ages 20-55) from the city of Tunis, divided into 4 socioeconomic groups (low, lower middle, higher middle, and high). We used objective and subjective socioeconomic measures and assessed cognitive and behavioral executive functioning through various tests, including the Stroop and Hayling tests, verbal fluency tasks, and the BRIEF questionnaire.

**Results:**

ANOVA analyses didn’t show global differences between groups, but Fisher Post Hoc tests revealed that participants from the highest socioeconomic group (group 4) performed better on several tasks. Group 4 showed faster processing times on Stroop tasks, better scores on the Digit Span Task, verbal fluency tasks, and the Modified Card Sorting Test, indicating a better processing speed and stronger cognitive flexibility and working memory. Behavioral executive measures also favored group 4 in areas such as working memory and task control.

**Conclusions:**

This study highlights the clear advantage of higher socioeconomic status in executive functioning. Further research could develop strategies to improve cognitive functioning and quality of life for individuals with a lower socioeconomic level.

**Disclosure of Interest:**

None Declared

